# Type 2 immunity in asthma

**DOI:** 10.1186/s40413-018-0192-5

**Published:** 2018-06-26

**Authors:** Marco Caminati, Duy Le Pham, Diego Bagnasco, Giorgio Walter Canonica

**Affiliations:** 1 Asthma Center and Allergy Unit, Verona University Hospital, Piazzale Scuro10, 37134 Verona, Italy; 2 Faculty of Medicine, University of Medicine and Pharmacy at Ho Chi Minh City, Ho Chi Minh City, Viet Nam; 3 University of Genoa Allergy and Respiratory Diseases, IRCCS San Martino Hospital, IST, University of Genoa, Genoa, Italy; 4 Personalized Medicine Clinic, Asthma & Allergy, Humanitas Clinical and Research Center, Humanitas University, Rozzano, Milan, Italy

**Keywords:** Innate immunity, Adaptive immunity, Type 2 inflammation, Asthma, Severe asthma, Allergic sensitization, Epithelial dysfunction, Biologicals

## Abstract

Type 2-immunity represents the typical adaptive response to allergen exposure in atopic individuals. It mainly involves Th2 cells and immunoglobulin E, as the main orchestrators of type 2-inflammation. Recently, it has been highlighted that allergens may be responsible for a Th2 response beside specific IgE activation and that a number of other environmental stimuli, such as viruses and pollutants, can trigger the same pattern of inflammation beyond atopy. Emerging data sustain a substantial role of the so-called epithelial dysfunction in asthma pathogenesis, both from anatomic and functional point of view. Furthermore an increasing amount of evidence demonstrates the relevance of innate immunity in polarizing a Th2 impaired response in asthmatic patients. Under this perspective, the complex cross-talking between airway epithelium, innate and adaptive immunity is emerging as a major determinant of type 2-inflammation beyond allergens.

This review will include an update on the relevance of dysregulation of innate and adaptive type 2-immunity in asthma pathogenesis, particularly severe asthma, and on the role of the allergens that are associated with severe asthma. Type 2-immunity also will be reviewed in the light of the current and upcoming targeted treatments for severe asthma.

## Key points

- Asthma pathogenesis can be explained as the consequence of an epithelial barrier dysfunction, which entails loss of anatomic integrity and impaired functional, mainly immunological, competence, the last aspect being strictly connected with innate immunity.
- In atopic individuals allergen exposure promotes the activation of Th2 cytokines, particularly IL-4, IL-5, IL-9, and IL-13, which orchestrate and amplify type-2 response.
- Eosinophil activation is the key-step of type 2-inflammatory cascade, not only, but mostly in the late allergic response, and eosinophils exert a central role in asthma pathogenesis through the release of four main proteins and a number of mediators which sustain amplification of allergic response and remodelling.
- Sensitization to several specific allergens, including house dust mite (HDM), fungi, pollen, animal dander and cockroach, are associated with a more severe phenotype of allergic asthma.
- A number of allergens, including HDM and moulds, are able to trigger a type 2 immune response in the absence of specific IgE antibodies, by activating airway epithelial Cells (AECs) via pathogen recognition receptor (PAR) and toll-like receptors (TLR) to produce TSLP, IL-25 and IL-33. The same pattern can be activated by allergens proteases and oxidative damage.
- Mainly in response to selective TLR (TLR 4, 5 and 9 particularly) stimulation and to injury operated by environmental stimuli (viruses and pollutants), the epithelial cells produce master regulatory cytokines, including thymic stromal lymphopoietin (TSLP), IL-25 and IL-33, which stimulate Th2 cells and type 2 innate lymphoid cells (ILC2) to produce Th2 cytokines.
- ILC2 orchestrate a second-line adaptive type 2 immune response, a chronic eosinophilic airways inflammation in patients with asthma, particularly severe asthma, and an altered tissue repair processes responsible for airway remodelling, which further sustains the epithelial physical inefficiency.
- In the light of the emerging pathogenic role of epithelial dysfunction and innate immunity, even type 2-inflammation should be no more considered as a consequence of allergenic stimuli only, but the result of a complex cross-talking between airway epithelium, innate and adaptive immunity.
- Th2 immunity actors represent the selective target of a number of current and upcoming biological treatments. Innate immunity, particularly TSLP, is under investigation as a novel target for innovative drugs.

## Background: type 2 immune response

Traditionally, type 2-immunity is triggered by allergens or parasitic infection and characterized by the differentiation of naïve T CD4+ cells towards Th2 effector cells, which is typically associated with IgE production, eosinophilia and mast cell activation. The keystone cytokines in type 2 immune response include interleukin (IL) 4, IL-5, IL-9, and IL-13 [1, 2]. IL-4 is crucial for the differentiation of naïve Th0 cells to Th2 cells, which in turn induce isotype switching to IgE production. Specific IgE antibodies bind to their high affinity receptors FceRI on the surface of basophils or mast cells, leading to the sensitization of those cells. IL-5 and IL-9 are responsible for the activation and recruitment of eosinophils and mast cells, while IL-13 induces goblet cell hyperplasia, mucus hyper-secretion and airway hyper-responsiveness [3, 4]. Nevertheless, the initiation of immune response is believed to be triggered by the innate immune cells located at the epithelium of the skin, lung, or gut. Injured epithelial cells produce master regulatory cytokines, including thymic stromal lymphopoietin (TSLP), IL-25 and IL-33. Subsequently, those cytokines stimulate Th2 cells and type 2 innate lymphoid cells (ILC2) to produce Th2 cytokines. Furthermore, IL-33 and TSLP could directly activate mast cells, while TSLP stimulates dendritic cells to induce a Th2-like process.

In infection, Th2 immune response provides an effective protection against helminths. Th2-induced IgE antibody production, mucus secretion, and mast cell activation could facilitate worm expulsion; while eosinophils recruited by Th2 response directly kill larva [5]. Additionally, Th2 immune response is known to counteract Th1 response in the microbicidal reactions, which could help limit the tissue damage induced by Th1-mediated inflammation [6]. Nevertheless, the polarization of immune responses excessively toward Th2 immunity leads to atopic disorders including asthma, allergic rhinitis, and atopic dermatitis.

Studies have shown an important role of Th2 immunity in the immunopathology of asthma, which could determine the severity of the disease. This review will describe the dysregulation of innate and adaptive type 2 immunity in severe asthma, the roles of the allergens that are associated with severe asthma and targeting type 2 immune response in treatment of severe asthma.

## Type 2-inflammation in the frame of unbalanced adaptive immunity

Type 2-inflammation is traditionally sustained by Th2 cells and immunoglobulin E (IgE), as the main orchestrators of type 2 adaptive immunity [7]. In atopic individuals, as a consequence of allergen exposure, dendritic cells (DCs) acquire soluble protein allergen and reach secondary draining lymph nodes, where antigens are presented and naïve T cells polarize into either adaptive Th2 cells or T_FH_ cells. Through specific signalling pathways, Th2 cells move toward the site of inflammation and produce Th2 cytokines, particularly IL-4, IL-5, IL-9, and IL-13, which orchestrate and amplify type-2 response [1, 7]. Tissue eosinophilia and mast cell hyperplasia are independently regulated by IL-5 and IL-9, while IL-13 is responsible for goblet cell proliferation as well as mucin and mucus production, and airways hyper-reactivity (AHR) [4, 8]. T_FH_ cells are able to induce IgE production by interacting with antigen-specific B cells. Allergen-specific IgE crosslinking on basophils and mast cells high-affinity Fcε receptor 1 (FcεR1) activates and releases through degranulation a number of cytokines and mediators which further sustain type-2 response [9, 10]. In fact, mast cells and basophils serve as specified effectors in type 2 immunity response under the influence of the type-2 cytokines environment in local tissue [7]. Cytosolic granules in mast cells contain cytokines (IL-4, IL-5, IL-6, and IL-13), biogenic amines (histamine and serotonin), serglycin, proteoglycans, mast cell-derived proteases (chymase and tryptase), and lipid mediators (platelet-activating factor [PAF], leukotrienes, prostaglandins, and sphingolipids). Recruitment of inflammatory cells, eosinophils in particular, smooth muscle constriction, and increased vascular permeability directly depend on mast cells mediators’ release [1, 11].

Basophils sustain the late phase of allergic response, by promoting eosinophilic inflammation and mucus production. In comparison with mast cells, ILC2s, and eosinophils, they produce relatively high levels of IL-4, thus acting as major Th2 differentiation promoters, including ILC2 activation [12, 13].

Eosinophil activation is the key-step of type 2-inflammatory cascade, not only but mostly in the late allergic response, and eosinophils exert a central role in asthma pathogenesis through the release of four main proteins retained in their cytoplasmic granules and a number of mediators [14]. Eosinophil cationic protein (ECP) and eosinophil protein X (EPX), also called eosinophil-derived neurotoxin (EDN), are mainly characterized by cytotoxic properties and ribonuclease activity. ECP also binds calcium-sensing receptor (CSR) on smooth muscle cells. Eosinophil peroxidase (EPO) EPO serves in catalysing the reaction of hydrogen peroxide with halogens to hypohalides and, like the major basic protein (MBP), promotes cytotoxic processes. MBP also binds bronchial muscarinic M2 receptor [14].

Furthermore, eosinophils produce cytokines (mainly IL-1b, IL-6, IL-8 and IL-4), lipid mediators and oxygen radicals. Eosinophil activation results in dendritic cells chemotactic activity, endothelial cells damaging, inhibition of muscarinic receptors, altered repair processes and induction of fibrosis, leading to airway hyperactivity and wall remodelling [15, 16]. All of these effects have direct implications in asthma control impairment and exacerbations.

**Fig. 1:**
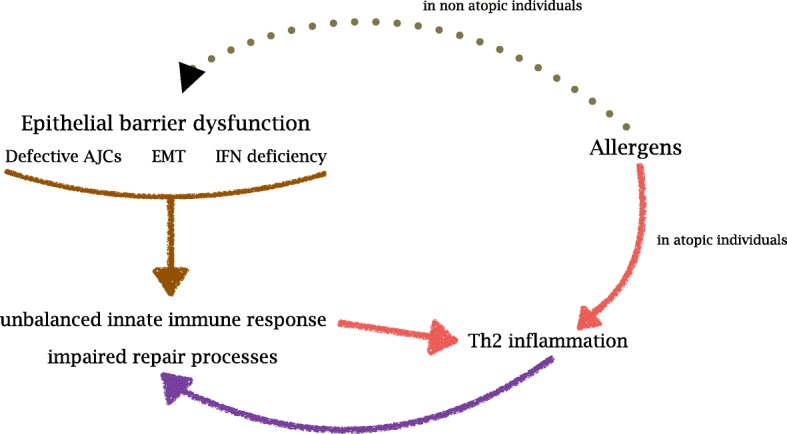
Synthetic overview of asthma Th2 inflammation main determinants. EMT: epithelial-mesenchimal transition; AJC: apical junctional complexes

Recently, an increasing amount of evidence has demonstrated that allergens can activate a Th2 response beside specific IgE involvement and that a number of other environmental stimuli can trigger the same pattern of inflammation [17, 18]. In light of the emerging pathogenic role of epithelial dysfunction and innate immunity, even type 2-inflammation should be considered no more as a consequence of allergenic stimuli only but the result of a complex cross-talking between airway epithelium, innate and adaptive immunity [19] (Fig. 1).

## Type 2-inflammation in the frame of unbalanced innate immunity

According to the most recent evidence, asthma pathogenesis could be synthetically explained as the consequence of an epithelial barrier dysfunction [17, 20] (Fig. 1), which is, after all, the common background of allergic/type 2 respiratory and skin diseases [21]. Epithelial barrier efficiency implies two main aspects: anatomic integrity and functional, mainly immunological, competence, the last aspect being strictly connected with innate immunity [20]. Under the anatomic perspective, the main problem with the epithelial barrier is that asthmatics produce defective apical junctional complexes (AJCs). Apical tight junctions and underlying adherens junctions [21] form AJCs, exerting a pivotal role in the physical integrity and functional stability of airways mucosa. Such a condition implies: an extremely weak physical defence against environmental stimuli such as viruses, allergens, and pollutants; and impaired epithelial wound repair responses. This scenario is mainly due to genetic predisposition, including primary genetic, secondary genetic (e.g. somatic mutations), or epigenetic (e.g. altered miRNA, histone acetylation/deacetylation, or DNA CpG methylation) [20, 22]. A model known as epithelial-mesenchymal transition (EMT) has been recently proposed as one of the determinants of the epithelial barrier dysfunction and epithelial-mesenchymal signalling dysregulation [17, 23]. A loss of E-cadherin would explain the impaired differentiation of epithelial cells into a mesenchymal phenotype, at embryogenesis level. Although still controversial, it may account for impaired repair processes and airway remodelling in asthmatics.

Smoke as well can be responsible for tight junctions inefficiency [17, 24]. Data coming from in vitro studies have confirmed that respiratory anatomic barrier impairment is independent of the presence of inflammatory cells or mediators, suggesting that epithelial injury plays a causal role in the disease onset, not only as a consequence of a longstanding inflammation [20, 25].

From a functional point of view, primary IFN production deficiency in response to common respiratory viruses such as rhinovirus might be responsible for a kind of aberrant differentiation of dendritic cells which become quite inefficient in anti-microbial protection whilst activate pro-allergic pathways [26, 27]. In fact, the lack of IFN β results in viral replication, cell cytotoxicity, and mediator release associated with non-protective inflammation. Such a condition may account for the association between frequent rhinovirus exposures in the lower respiratory tract and multiple allergen sensitization early in life [28, 29]. Also, a defective anti-oxidant pathway, including superoxide dismutase and glutathione peroxidase has been described in asthmatic subjects [30].

As a consequence of the impact of environmental agents usually excluded from the exposure to the second line immunity by the epithelial physical and functional barrier, a number of impaired epithelial signalling pathways take place. The disordered response includes a Th2 oriented unbalanced inflammation, and an exaggerated response to injury [31, 32]. The secretion of a number of growth factors leads to altered tissue repair processes and is responsible for airway remodelling, which further sustains the epithelial physical inefficiency.

Mainly in response to selective toll-like receptor (TLR 4, 5 and 9 particularly) stimulation and to injury, the epithelial cells, oriented by a network of transcription factors including FoxA2 and Spdef, start producing key cytokines such as chemokine (C-C motif) ligand (CCL) 17 and CCL22 that exert most of Th2-type T-cell chemotactic activity by interacting with the common CCR4 receptor [21, 33]. Other master regulatory cytokines produced by the epithelial cells include TSLP, IL-25 and IL-33, as well as IL-13, TARC, RANTES, eotaxins, and MCP-3 [21, 34, 35]. As a major effect, those molecules interact with ILC2 to produce Th2 cytokines and are able to control mucous cell differentiation. Recently, an increasing amount of evidence supports the key role of ILC2 in orchestrating a second-line adaptive type 2 immune response, a chronic eosinophilic airways inflammation in patients with asthma, particularly severe asthma, and the consequent airways remodelling [7, 36, 37]. Furthermore, they seem involved in a kind of steroid resistance, especially under the influence of TSLP and IL-7 [34]. Besides ILC2, epithelial-derived IL-33 and TSLP could directly activate mast cells and eosinophils, while TSLP stimulates dendritic cells to induce a Th2-like process [38, 39].

Emerging data suggest that mast cells, when directly activated by plant-derived allergen proteases, further promote IL-33 and release several cytokines, including IL-2 and IL-4. This results in altered expansion of Treg cells and dysregulated expression of their GATA3 and Th2-type cytokines (IL-5, IL-13), which impairs their regulatory and suppressive function towards Th2 inflammation [7, 38].

Dendritic cells (DCs), mainly monocytic DCs, substantially contribute to Th2 polarization even in the absence of specific allergen stimulation [33, 40, 41]. Stimulation of DCs pattern-recognition receptors, such as TLR, by high dose of protease allergen results in production of CCL17 and CCL22, which recruits effector Th2 cells to produce IL-5 and IL-13 and further sustains eosinophilic inflammation.

## Allergen sensitization and type 2 immune response: Beyond IgEs

Studies have shown that sensitization to several specific allergens, including house dust mite (HDM), fungi, pollen, animal dander, and cockroach, are associated with a more severe phenotype of allergic asthma [42]. Allergen-derived particles with a diameter ≤5 μm, such as HDM particles, fungal spores, and animal dander, could penetrate deeply to the lower airways, thereby more likely causing asthma [43].

HDM is one of the most common aeroallergens inducing sensitization in 85% patients with asthma [44]. HDM can activate immune system by not only mite-derived allergens (including proteases and other allergenic determinants) but also components originated from the environment (including LPS from bacteria, β-glucan and chitin from fungi) [45]. HDM-derived allergens can provoke a Th2 response leading to the production of specific IgE antibody, eosinophil and mast cell activation; while microbial contaminants in HDM particles activate airway epithelial cells (AECs) via pathogen recognition receptor (PRR) and TLR to produce TSLP, IL-25 and IL-33, which amplify type 2 immune response induced by HDM [18, 45]. Nevertheless, HDM can activate AECs to produce IL-6 and IL-8, leading to an airway neutrophilia [46, 47]. In the airway epithelium of severe asthmatic subjects, HDM could simulate the proliferation of bronchial smooth muscle cells, which could be abolished by montelukast [45]. Recently, HDM was found to directly impair the airway epithelium by causing oxidative damage, DNA double-strand break and apoptosis in the AECs, which enhance the HDM-induced airway inflammation in asthma [48, 49]. Consequently, HDM is one of the most powerful allergens that could activate both innate and adaptive type 2 immune responses, thereby increasing asthma severity in sensitized individuals. However, the underlying mechanism of HDM-induced airway inflammation and remodeling in asthma still remains to be investigated.

Sensitization to certain fungi has been associated with increased asthma severity, mortality, hospitalization, and intensive care admissions in adults [50]. There are 3 distinct ways of fungal allergen exposure associated with severe asthma: 1) inhalation of spores or hyphae in the airbone (e.g. *Alternaria*) that induce airway hyperresponsiveness in sensitized individuals; 2) colonization of fungi (e.g. *Aspergillus*, *Penicillium* and *Candida*) in the airways that produces a persistently allergenic stimulus; 3) fungal colonization outside of the airways (e.g. dermatophyte infection of the skin or nails with *Trichophyton*) that can trigger immediate hypersensitivity [51]. The fungal allergens can not only induce type 2-immune response by their allergenic activity, but also trigger the production of IL-6, and IL-8 from the AECs as well as Th17 differentiation [52, 53], leading to a neutrophilic inflammation of the airways. A wide spectrum of effects of fungal allergens on the immune system enhances the airway inflammation, leading to an increased asthma severity.

There is abundant evidence showing the associations of tree, grass, and weed pollen sensitization with severe asthma [43]. The most relevant sources of allergenic pollen worldwide include trees belonging to the *Fagales*, *Oleacea*, and *Cupressacecae *families; grasses of the *Pooideae*, *Chloridoideae* and *Panicoideae* families; weeds of the *Amaranthaceae*, *Asteraceae*, and *Urticaceae* families [54–56]. Recent findings showed that environmental changes and air pollutants significantly enhance the allergenic activity and biologic effects of the pollen on type 2 immune response, which could increase the severity of pollen-induced airway inflammation in asthma [57]. Additionally, more evidence has implicated that thunderstorm is a risk factor for severe asthma attacks in sensitized subjects [58]. Consequently, an early warning system for the outbreaks of thunderstorm asthma by accompanying meteorology and pollen counts has been evaluated recently but has shown limited practical applications and needs to be optimized [58–60].

Other indoor allergens that are associated with increased asthma risk include animal dander and cockroach. The presence of pets at home and sensitizations to cat or dog allergens were also found to be associated with severe asthma [61], while sensitization to cockroach allergens was associated with more asthma symptoms and more school missed due to asthma in children [62]. Nevertheless, the underlying mechanisms of how those allergens enhance asthma risk and severity are not completely understood and need to be further investigated.

Although allergen sensitizations are well-known to be associated with asthma, a number of studies reported a lower frequency of allergen sensitizations, obtained by skin prick tests (SPT) or specific serum IgE measurement, in patients with severe asthma compared to those with moderate or mild asthma [42, 63]. These findings could be explained by a local allergen sensitization, leading to a production of specific IgE antibodies in the airways, despite negative response in SPTs and low level of serum specific IgE [64]. Additionally, T-lymphocyte activation and airway eosinophilia could exist in patients with non-allergic asthma, suggesting that some factors could induce a type 2-immune response without inducing specific sensitizations [65, 66]. LPS-containing allergens, such as HDM, could stimulate innate immune cells to produce TSLP, IL-25 and IL-33, which could provoke a type 2 immune response [1]. Proteases in allergens are also able to activate proteinase-activated receptor 2 (PAR2) expressed on AECs, inducing the production of TSLP and IL-33 [53, 67]. Those cytokines could directly activate ILC2 to produce a large amount of IL-5 and IL-13, which cause an airway eosinophilia [68].

Taken together, allergens could induce type 2 immune response in various ways to maintain and/or amplify the airway inflammation and remodelling in asthma, which is associated with severe asthma. Understanding the mechanisms of allergen-induced immune response help select a target therapy in management of severe asthma.

## Type 2-immunity as a target of precision treatments

The awareness of two different types of inflammation (Th2-high and Th2-low), and that Th2 type is frequently involved in several kinds of severe asthma, has driven research to look for drugs targeting Th2 cytokines or their production mechanisms [69, 70] (Table 1). Principal Th2 phenotype cells are eosinophils. Their differentiation, maturation, and survival processes depend on several cytokines, mainly interleukin-5 (IL-5), IL-3 and granulocyte-macrophage colony-stimulating factor (GM-CSF). Eosinophils, moreover, promote the synthesis and release of IL-2, IL-3, IL-4, IL-5, IL-10, IL-12, IL-13, IL-16, IL-25, transforming growth factor (TGF) alpha and beta, CCL5, CCL11 and 13 [71, 72]. A GINA document [73] includes in therapeutic last step two monoclonal antibodies for severe uncontrolled asthmatic patients. Omalizumab binds circulating IgEs in blood and interstitial space also reducing basophils, mast cells and dendritic cells, [74, 75], reducing inflammation prompted by mediators produced from mast cells and a decreased recruitment of eosinophils in airways [75–78]. The efficacy of Omalizumab on symptoms [79], exacerbations rate [80], reduction of oral corticosteroids intake [81, 82], and modulation of bronchial remodelling process [83], together with a good safety profile, both in adults and children [84], has been clearly demonstrated. Real life observations show comparable results with trials [82, 85–88] with significant improvement in FEV1 after 4 years of therapy with [89] and improvement in quality of life (QoL) [90]. Mepolizumab, recently added in the GINA guidelines is an anti-IL-5 humanized antibody [91]. Several studies demonstrate that people with high levels of eosinophils and frequent exacerbations better respond to this therapy. One of the last trials demonstrated that mepolizumab provided a significant reduction of the exacerbations incidence rate in patients with blood mean eosinophilia of 654 cells/μL and [92]. In a Cochrane meta-analysis two studies reported a significant decrease in the exacerbation rate in asthmatic patients with high level of blood eosinophils (>300 μL). Four studies with serum eosinophils heterogeneity levels have shown a non-significant difference between mepolizumab and placebo in terms of reduction of exacerbations, probably due to the poor phenotypization of patients [93].

**Table 1:** Biologicals targeting Th2 inflammation and their main effects

Drug	Exacerbations	Lung Function	QoL
Mepolizumab (ANTI IL-5)	Reduction [92, 94–96]	Increase [92, 96]; no variation in FEV1 [94, 96]	Increase [94, 97, 98], no variation in QoL [96]
Reslizumab (ANTI IL-5)	Reduction [100, 101]	Increase of FEV1 [102]	Increase of QoL [100]
Benralizumab (ANTI IL-5Ra)	Reduction [103, 106–109]	Increase of FEV1 [107–109]; no variation [106]	Increase of QoL [107–109]; no variation [106]
Pitrakinra (ANTI IL-4)	Reduction in homozygous for the rs8832 common G allele, rs1029489, and the intronic SNPs rs3024585, rs3024622 and rs4787956 [111]	Reduction [111]	n.a.
Dupilumab (ANTI IL-4rα)	Reduction [112, 113]	Increase of FEV1 [112, 113]	Increase of QoL [14, 112]
Lebrikizumab (ANTI IL-13)	Reduction in high periostin group [115]	Increase of FEV1 in high periostin group [115, 116], response in high periostin, IgE and eosinophils group, [117], no variation [118]	No variation [115]
Tralokinumab (ANTI IL-13)	Reduction in high-periostin and high-DPP-4 groups [119]	Increase in high-periostin and high-DPP-4 groups [119]	Increase of QoL [120]
Tezepelumab (ANTI TSLP)	Reduction [122]	Increase [122]	Increase at medium and high dosage [122]

The main clinical trials assessing the efficacy of mepolizumab [92, 94–98] reported a reduction of exacerbations in treated patients, a good safety profile with overall five deaths, three in active group [96], two in placebo [97, 98]. The most frequently adverse events (AEs) were nasopharyngitis, upper respiratory tract infection and headache, all with similar percentage both in active and placebo group. MUSCA study evaluated the effect on health related quality of life (HEQOL) with, a significant change in favor of patients who had received mepolizumab [92]. Recently approved by the Food and Drug Aministration (FDA) in its intravenous formulation (https://www.accessdata.fda.gov/drugsatfda_docs/appletter/2016/761033orig1s000ltr.pdf), reslizumab is an IgG4/k humanized antibody targeting IL-5 [99]. Also, reslizumab has exhibited a better efficacy in hypereosinophilic severe asthmatic patients with at least 400 eosinophils/μL [100–102]. Regarding safety, two anaphylactic reactions, responsive to standard treatment, a case of pneumonia, one of worsening of asthma, upper respiratory tract infections, and nasopharyngitis, all in the reslizumab group, have been reported [100]. In addition, IL-5 receptor alpha has been targeted in the management of severe asthma. Benralizumab is a biological drug targeting IL-5R*α* subunit [103], whose action is directed both on eosinophils and on basophils [104]. A peculiarity of this drug is its antibody-dependent cell-mediated cytotoxicity (ADCC) effect [103], mediated by its action on NK cells [105]. As well as mepolizumab and reslizumab, benralizumab demonstrates a good efficacy and safety profile [106, 107]. In the CALIMA and SIROCCO trials significant reduction of exacerbations in people who assume drugs compared with placebo both in 4 weeks administration than in 8 weeks has been evidenced [108, 109]. The last schedule is associated with a better efficacy in terms of reduction of exacerbations, asthma symptoms score and lung function, according to the results shown by the registrations trials, particularly CALIMA and SIROCCO. The mechanisms underlying this trend are not yet completely clear. The involvement of the ADCC pathway, which probably entails a different timing in comparison with an IL-blockage system, may account for it.

IL-4 and IL-13 have been analysed as well. IL-4 pathway could be inhibited by either blocking IL-4 or the IL-4/IL-13 receptor. Pascolizumab, a humanized monoclonal antibody (mAb) blocking IL-4, was found to be well tolerated; however, its effect on asthma symptoms and IgE level reduction was insignificant [110]. A mutated IL-4 protein, pitrakinra, acts as an antagonist binding to IL-4 receptor alpha chain, thereby inhibiting both IL-4 and IL-13 pathway [111]. In a clinical trial, inhaled pitrakinra could reduce significantly asthma exacerbation and the effect was depedent on the polymorphisms of IL-4Rα gene [111]. Dupilumab, another monoclonal antibody to IL-4Rα, was effective in reducing asthma exacerbation and several biomarkers, including FeNO, eotaxin-3 as well as thymus and activation-regulated cytokine (TARC) [112, 113]. IL-13 is as important as IL-4 in type 2 immune response in asthma. Anrukinzumab is a humanized anti-IL-13 mAb, which has been tested in patients with asthma and ulcerative colitis in a phase II study [114]. Lebrikizumab, a IgG4 humanized mAb, binds to soluble IL-13 and block IL-13 pathway, was effective in improving lung function and reducing asthma exacerbation rates and FeNO levels in asthmatic patients with high serum periostin level [115–118]. Tralokinumab, an IgG4 humanized mAb that binds to IL-13, showed a good tolerance and safety profile, and could improve quality of life. However, no significant effect of tralokinumb on asthma exacerbation rates was observed [119, 120]. In addition, tralokinumab is currently being investigated in patients with idiopathic pulmonary fibrosis due to the crucial role of IL-13 in airway remodeling [121]. More recently stromal lymphopoietin (TSLP), has been evaluated as pharmaceutical target. Tezepelumab, has been evaluated in three different dosage (70 or 210 every 4 weeks and 250 mg every 8) administrated to asthmatic patients, with the aim to evaluate the difference of exacerbations. The primary end point, the annual exacerbations rate result significantly higher in placebo (0.67) compared to treated patients (0.26; 0.19; 0.22) with a statistically significant reduction in who assumed drug. Also lung function was found to be higher in treated patients than placebo [122].

## Conclusions

Th2 immunity exerts a pivotal role in asthma pathogenesis, beyond allergic sensitization. In fact, not only specific IgE in sensitized individuals but also many other environmental stimuli, such as viruses and pollutants, can trigger a Th2 response. Furthermore, a number of allergens such as HDM and moulds are able to activate both innate and adaptive type 2 immune reaction even in the absence of specific IgE antibodies. An increasing amount of evidence supports the relevance of airways, particularly bronchial epithelium dysfunction, as the predisposing condition of such impaired response. Epithelial barrier efficiency implies two main aspects: anatomic integrity and functional, mainly immunological, competence, the last aspect being strictly connected with innate immunity. Under this perspective the Th2 polarization is the result of a complex cross talking between airway epithelium, and innate and adaptive immunity. It entails major clinical implications in terms of preventive and therapeutic options. Particularly, innate response can be considered as a new target for innovative selective treatments.

## Abbreviations

AEC: Airways epithelial cells
DCs: Dendritic cells
IgE: Immunoglobulin E
IL: Interleukin
ILC2: Innate lymphoid cells
T_FH_: Follicular helper t cells
TSLP: Thymic stromal lymphopoietin

## References

[1] Oliphant CJ, Barlow JL, McKenzie AN (2011). Insights into the initiation of type 2 immune responses. Immunology.

[2] Wynn TA (2015). Type 2 cytokines: mechanisms and therapeutic strategies. Nat Rev Immunol.

[3] Fahy JV (2015). Type 2 inflammation in asthma—present in most, absent in many. Nat Rev Immunol..

[4] Zhu Z, Homer RJ, Wang Z, Chen Q, Geba GP, Wang J (1999). Pulmonary expression of interleukin-13 causes inflammation, mucus hypersecretion, subepithelial fibrosis, physiologic abnormalities, and eotaxin production. J Clin Invest.

[5] Finkelman FD, Urban JF (2001). The other side of the coin: the protective role of the Th2 cytokines. J Allergy Clin Immunol.

[6] Berger A (2000). Th1 and Th2 responses: what are they?. BMJ.

[7] Kubo M (2017). Innate and adaptive type 2 immunity in lung allergic inflammation. Immunol Rev.

[8] Zhu J, Yamane H, Paul WE (2010). Differentiation of effector CD4 T cell populations. Annu Rev Immunol.

[9] Harada Y, Tanaka S, Motomura Y (2012). The 3′ enhancer CNS2 is a critical regulator of interleukin-4-mediated humoral immunity in follicular helper T cells. Immunity.

[10] Ballesteros-Tato A, Randall TD, Lund FE, Spolski R, Leonard WJ, León B (2016). T follicular helper cell plasticity shapes pathogenic T helper 2 cell-mediated immunity to inhaled house dust mite. Immunity.

[11] Sawaguchi M, Tanaka S, Nakatani Y (2012). Role of mast cells and Ba-sophils in IgE responses and in allergic airway hyperresponsiveness. J Immunol.

[12] Siracusa MC, Kim BS, Spergel JM, Artis D (2013). Basophils and allergic inflammation. J Allergy Clin Immunol.

[13] Egawa M, Mukai K, Yoshikawa S (2013). Inflammatory monocytes recruited to allergic skin acquire an anti-inflammatory M2 phenotype via basophil-derived interleukin-4. Immunity.

[14] Amin K, Janson C, Bystrom J (2016). Role of eosinophil granulocytes in allergic airway inflammation Endotypes. Scand J Immunol.

[15] Doran E, Cai F, Holweg CTJ, Wong K, Brumm J, Arron JR (2017). Interleukin-13 in asthma and other eosinophilic disorders. Front Med (Lausanne).

[16] Yasukawa A, Hosoki K, Toda M, Miyake Y, Matsushima Y, Matsumoto T, Boveda-Ruiz D, Gil-Bernabe P, Nagao M, Sugimoto M, Hiraguchi Y, Tokuda R, Naito M, Takagi T, D’Alessandro-Gabazza CN, Suga S, Kobayashi T, Fujisawa T, Taguchi O, Gabazza EC (2013). Eosinophils promote epithelial to mesenchymal transition of bronchial epithelial cells. PLoS One.

[17] Holgate ST (2011). The sentinel role of the airway epithelium in asthma pathogenesis. Immunol Rev.

[18] Trian T, Allard B, Dupin I, Carvalho G, Ousova O, Maurat E (2015). House dust mites induce proliferation of severe asthmatic smooth muscle cells via an epithelium-dependent pathway. Am J Respir Crit Care Med.

[19] Hinks TS, Zhou X, Staples KJ, Dimitrov BD, Manta A, Petrossian T, Lum PY, Smith CG, Ward JA, Howarth PH, Walls AF, Gadola SD, Djukanović R (2015). Innate and adaptive T cells in asthmatic patients: relationship to severity and disease mechanisms. J Allergy Clin Immunol.

[20] Georas SN, Rezaee F (2014). Epithelial barrier function: at the front line of asthma immunology and allergic airway inflammation. J Allergy Clin Immunol.

[21] Lloyd CM, Saglani S (2015). Epithelial cytokines and pulmonary allergic inflammation. Curr Opin Immunol.

[22] Gon Y, Hashimoto S. Role of airway epithelial barrier dysfunction in pathogenesis of asthma. Allergol Int. 2018;67:12–17. 10.1016/j.alit.2017.08.011 28941636

[23] Loffredo LF, Abdala-Valencia H, Anekalla KR, Cuervo-Pardo L, Gottardi CJ, Berdnikovs S. Beyond epithelial-to-mesenchymal transition: common suppression of differentiation programs underlies epithelial barrier dysfunction in mild, moderate, and severe asthma. Allergy. 2017;72:1988–2004. 10.1111/all.13222 PMC5698119 28599074

[24] Aghapour M, Raee P, Moghaddam SJ, Hiemstra PS, Heijink IH. Airway epithelial barrier dysfunction in COPD: role of cigarette smoke exposure. 2018;58:157–69 10.1165/rcmb.2017-0200TR 28933915

[25] Fedorov IA, Wilson SJ, Davies DE, Holgate ST (2005). Epithelial stress and structural remodel- ling in childhood asthma. Thorax.

[26] Jackson DJ, Makrinioti H, Rana BM, Shamji BW, Trujillo-Torralbo MB, Footitt J, Del-Rosario J, Telcian AG, Nikonova A, Zhu J, Aniscenko J, Gogsadze L, Bakhsoliani E, Traub S, Dhariwal J, Porter J, Hunt D, Hunt T, Hunt T, Stanciu LA, Khaitov M, Bartlett NW, Edwards MR, Kon OM, Mallia P, Papadopoulos NG, Akdis CA, Westwick J, Edwards MJ, Cousins DJ, Walton RP, Johnston SL (2014). IL-33-dependent type 2 inflammation during rhinovirus-induced asthma exacerbations in vivo. Am J Respir Crit Care Med.

[27] Beale J, Jayaraman A, Jackson DJ, Macintyre JDR, Edwards MR, Walton RP, Zhu J, Man Ching Y, Shamji B, Edwards M, Westwick J, Cousins DJ, Yi Hwang Y, McKenzie A, Johnston SL, Bartlett NW (2014). Rhinovirus-induced IL-25 in asthma exacerbation drives type 2 immunity and allergic pulmonary inflammation. Sci Transl Med.

[28] Bosco A, Ehteshami S, Stern DA, Martinez FD (2010). Decreased activation of inflammatory networks during acute asthma exacerbations is associated with chronic airflow obstruction. Mucosal Immunol.

[29] Han H, Roan F, Ziegler SF (2017). The atopic march: current insights into skin barrier dysfunction and epithelial cell-derived cytokines. Immunol Rev.

[30] Omhair SA, Erzurum SC (2010). Redox control of asthma: molecular mechanisms and thera- peutic opportunities. Antioxid Redox Signal.

[31] Boulet LP. Airway remodeling in asthma: update on mechanisms and therapeutic approaches. Curr Opin Pulm Med. 2017; [Epub ahead of print] 10.1097/MCP.0000000000000441 29076828

[32] Loxham M, Davies DE (2017). Phenotypic and genetic aspects of epithelial barrier function in asthmatic patients. J Allergy Clin Immunol.

[33] Lee LM, Ji M, Sinha M, Dong MB, Ren X, Wang Y, Lowell CA, Ghosh S, Locksley RM, DeFranco AL (2016). Determinants of divergent adaptive immune responses after airway sensitization with ligands of toll-like receptor 5 or toll-like receptor 9. PLoS One.

[34] Liu S, Verma M, Michalec L, Liu W, Sripada A, Rollins D, Good J, Ito Y, Chu H, Gorska MM, Martin RJ, Alam R (2018). Steroid resistance of airway type 2 innate lymphoid cells from patients with severe asthma: the role of thymic stromal lymphopoietin. J Allergy Clin Immunol.

[35] Hu Y, Dong H, Zou M, Huang C, Luo L, Yu C, Chen J, Xie Z, Zhao H, Le Y, Zou F, Liu L, Cai S (2017). TSLP signaling blocking alleviates E-cadherin dysfunction of airway epithelium in a HDM-induced asthma model. Cell Immunol.

[36] Smith SG, Chen R, Kjarsgaard M, Huang C, Oliveria JP, O’Byrne PM, Gauvreau GM, Boulet LP, Lemiere C, Martin J, Nair P, Sehmi R (2016). Increased numbers of activated group 2 innate lymphoid cells in the airways of patients with severe asthma and persistent airway eosinophilia. J Allergy Clin Immunol.

[37] Larose MC, Archambault AS, Provost V, Laviolette M, Flamand N (2017). Regulation of eosinophil and group 2 innate lymphoid cell trafficking in asthma. Front Med (Lausanne).

[38] Chen CC, Kobayashi T, Iijima K, Hsu FC, Kita H. IL-33 dysregulates regulatory T cells and impairs established immunologic tolerance in the lungs. J Allergy Clin Immunol. 2017; 10.1016/j.jaci.2017.01.015 PMC5554091 28196763

[39] Bartemes KR, Kephart GM, Fox SJ, Kita H (2014). Enhanced innate type 2 immune response in peripheral blood from patients with asthma. J Allergy Clin Immunol.

[40] Tussiwand R, Everts B, Grajales-Reyes GE (2015). Klf4 expression in conventional dendritic cells is required for T helper 2 cell responses. Immunity.

[41] Halim TY, Steer CA, Matha L (2016). Group 2 innate lymphoid cells license dendritic cells to potentiate memory TH2 cell responses. Nat Immunol.

[42] The ENFUMOSA cross-sectional European multicentre study of the clinical phenotype of chronic severe asthma. European network for understanding mechanisms of severe asthma. Eur Respir J. 2003;22(3):470–7. 10.1183/09031936.03.00261903 14516137

[43] Lombardi C, Savi E, Ridolo E, Passalacqua G, Canonica GW (2017). Is allergic sensitization relevant in severe asthma? Which allergens may be culprit?. World Allergy Organ J.

[44] Gregory LG, Lloyd CM (2011). Orchestrating house dust mite-associated allergy in the lung. Trends Immunol.

[45] Jacquet A (2013). Innate immune responses in house dust mite allergy. ISRN Allergy.

[46] Adam E, Hansen KK, Astudillo Fernandez O, Coulon L, Bex F, Duhant X (2006). The house dust mite allergen Der p 1, unlike Der p 3, stimulates the expression of interleukin-8 in human airway epithelial cells via a proteinase-activated receptor-2-independent mechanism. J Biol Chem.

[47] Kauffman HF, Tamm M, Timmerman JA, Borger P (2006). House dust mite major allergens Der p 1 and Der p 5 activate human airway-derived epithelial cells by protease-dependent and protease-independent mechanisms. Clin Mol Allergy.

[48] Chan TK, Loh XY, Peh HY, Tan WN, Tan WS, Li N (2016). House dust mite-induced asthma causes oxidative damage and DNA double-strand breaks in the lungs. J Allergy Clin Immunol.

[49] Oida K, Einhorn L, Herrmann I, Panakova L, Resch Y, Vrtala S, Hofstetter G, Tanaka A, Matsuda H, Jensen-Jarolim E (2017). Innate function of house dust mite allergens: robust enzymatic degradation of extracellular matrix at elevated pH. World Allergy Organ J..

[50] Denning DW, O’Driscoll BR, Hogaboam CM, Bowyer P, Niven RM (2006). The link between fungi and severe asthma: a summary of the evidence. Eur Respir J.

[51] Kennedy JL, Heymann PW, Platts-Mills TA (2012). The role of allergy in severe asthma. Clin Exp Allergy.

[52] Hernandez-Santos N, Gaffen SL (2012). Th17 cells in immunity to Candida albicans. Cell Host Microbe.

[53] Kauffman HF, Tomee JF, van de Riet MA, Timmerman AJ, Borger P (2000). Protease-dependent activation of epithelial cells by fungal allergens leads to morphologic changes and cytokine production. J Allergy Clin Immunol.

[54] Ferreira F, Gadermaier G, Wallner M, Akdis CA, Agache I (2014). Tree pollens allergens. EAACI global atlas of allergy: EAACI.

[55] Kleine-Tebbe J, Davies J, Akdis CA, Agache I (2014). Grass pollen allergens. EAACI global atlas of allergy: EAACI.

[56] Weber RW, Akdis CA, Agache I (2014). Weed pollen allergens. EAACI global atlas of allergy: EAACI.

[57] Lee SI, Pham le D, Shin YS, Suh DH, Park HS (2014). Environmental changes could enhance the biological effect of hop J pollens on human airway epithelial cells. J Allergy Clin Immunol.

[58] D’Amato G, Vitale C, D’Amato M, Cecchi L, Liccardi G, Molino A (2016). Thunderstorm-related asthma: what happens and why. Clin Exp Allergy.

[59] D’Amato G, Cecchi L, Annesi-Maesano I (2012). A trans-disciplinary overview of case reports of thunderstorm-related asthma outbreaks and relapse. Eur Respir Rev.

[60] De Linares C, Diaz de la Guardia C, Nieto Lugilde D, Alba F (2010). Airborne study of grass allergen (lol p 1) in different-sized particles. Int Arch Allergy Immunol.

[61] Tunnicliffe WS, Fletcher TJ, Hammond K, Roberts K, Custovic A, Simpson A (1999). Sensitivity and exposure to indoor allergens in adults with differing asthma severity. Eur Respir J.

[62] Gruchalla RS, Pongracic J, Plaut M, Evans R, Visness CM, Walter M (2005). Inner City asthma study: relationships among sensitivity, allergen exposure, and asthma morbidity. J Allergy Clin Immunol.

[63] Moore WC, Bleecker ER, Curran-Everett D, Erzurum SC, Ameredes BT, Bacharier L (2007). Characterization of the severe asthma phenotype by the National Heart, Lung, and Blood Institute’s severe asthma research program. J Allergy Clin Immunol.

[64] Mouthuy J, Detry B, Sohy C, Pirson F, Pilette C (2011). Presence in sputum of functional dust mite-specific IgE antibodies in intrinsic asthma. Am J Respir Crit Care Med.

[65] Bentley AM, Menz G, Storz C, Robinson DS, Bradley B, Jeffery PK (1992). Identification of T lymphocytes, macrophages, and activated eosinophils in the bronchial mucosa in intrinsic asthma. Relationship to symptoms and bronchial responsiveness. Am Rev Respir Dis.

[66] Del Giacco SR, Bakirtas A, Bel E, Custovic A, Diamant Z, Hamelmann E, Heffler E, Kalayci Ö, Saglani S, Sergejeva S, Seys S, Simpson A, Bjermer L (2017). Allergy in severe asthma. Allergy.

[67] Snelgrove RJ, Gregory LG, Peiro T, Akthar S, Campbell GA, Walker SA (2014). Alternaria-derived serine protease activity drives IL-33-mediated asthma exacerbations. J Allergy Clin Immunol.

[68] van Rijt L, von Richthofen H, van Ree R (2016). Type 2 innate lymphoid cells: at the cross-roads in allergic asthma. Semin Immunopathol.

[69] De Ferrari L, Chiappori A, Bagnasco D, Riccio AM, Passalacqua G, Canonica GW (2016). Molecular phenotyping and biomarker development: are we on our way towards targeted therapy for severe asthma?. Expert Rev Respir Med.

[70] Fajt ML, Wenzel SE (2015). Asthma phenotypes and the use of biologic medications in asthma and allergic disease: the next steps toward personalized care. J Allergy Clin Immunol.

[71] George L, Brightling CE (2016). Eosinophilic airway inflammation: role in asthma and chronic obstructive pulmonary disease. Ther Adv Chronic Dis.

[72] Davoine F, Lacy P (2014). Eosinophil cytokines, chemokines, and growth factors: emerging roles in immunity. Front Immunol.

[73] Global Initiative for Asthma (2017). Global Strategy for Asthma Management and Prevention.

[74] Chang TW, Shiung YY (2006). Anti-IgE as a mast cell-stabilizing therapeutic agent. J Allergy Clin Immunol.

[75] Kallieri M, Papaioannou AI, Papathanasiou E, Ntontsi P, Papiris S, Loukides S (2017). Predictors of response to therapy with omalizumab in patients with severe allergic asthma - a real life study. Postgrad Med.

[76] Massanari M, Holgate ST, Busse WW, Jimenez P, Kianifard F, Zeldin R (2010). Effect of omalizumab on peripheral blood eosinophilia in allergic asthma. Respir Med.

[77] Djukanovic R, Hanania N, Busse W, Price D (2016). IgE-mediated asthma: new revelations and future insights. Respir Med.

[78] Yalcin AD, Celik B, Yalcin AN (2016). Omalizumab (anti-IgE) therapy in the asthma-COPD overlap syndrome (ACOS) and its effects on circulating cytokine levels. Immunopharmacol Immunotoxicol.

[79] Busse W, Corren J, Lanier BQ, McAlary M, Fowler-Taylor A, Cioppa GD, van As A, Gupta N (2001). Omalizumab, anti-IgE recombinant humanized monoclonal antibody, for the treatment of severe allergic asthma. J Allergy Clin Immunol.

[80] Teach SJ, Gill MA, Togias A, Sorkness CA, Arbes SJ, Calatroni A, Wildfire JJ, Gergen PJ, Cohen RT, Pongracic JA, Kercsmar CM, Khurana Hershey GK, Gruchalla RS, Liu AH, Zoratti EM, Kattan M, Grindle KA, Gern JE, Busse WW, Szefler SJ (2015). Preseasonal treatment with either omalizumab or an inhaled corticosteroid boost to prevent fall asthma exacerbations. J Allergy Clin Immunol.

[81] Rodrigo GJ, Neffen H (2015). Systematic review on the use of omalizumab for the treatment of asthmatic children and adolescents. Pediatr Allergy Immunol.

[82] Humbert M, Beasley R, Ayres J, Slavin R, Hébert J, Bousquet J, Beeh KM, Ramos S, Canonica GW, Hedgecock S, Fox H, Blogg M, Surrey K (2005). Benefits of omalizumab as add-on therapy in patients with severe persistent asthma who are inadequately controlled despite best available therapy (GINA 2002 step 4 treatment): INNOVATE. Allergy.

[83] Roth M, Zhao F, Zhong J, Lardinois D, Tamm M (2015). Serum IgE induced airway smooth muscle cell remodeling is independent of allergens and is prevented by Omalizumab. PLoS One.

[84] Hanania NA, Alpan O, Hamilos DL, Condemi JJ, Reyes-Rivera I, Zhu J, Rosen KE, Eisner MD, Wong DA, Busse W (2011). Omalizumab in severe allergic asthma inadequately controlled with standard therapy: a randomized trial. Ann Intern Med.

[85] Pace E, Ferraro M, Bruno A, Chiappara G, Bousquet J, Gjomarkaj M (2011). Clinical benefits of 7 years of treatment with omalizumab in severe uncontrolled asthmatics. J Asthma.

[86] Normansell R, Walker S, Milan SJ, Walters EH, Nair P. Omalizumab for asthma in adults and children. Cochrane Database Syst Rev. 2014;13(1):CD003559. 10.1002/14651858.CD003559.pub4 PMC10981784 24414989

[87] Alhossan A, Lee CS, MacDonald K, Abraham I (2017). “Real-life” effectiveness studies of Omalizumab in adult patients with severe allergic asthma: meta-analysis. J Allergy Clin Immunol Pract.

[88] Chen H, Eisner MD, Haselkorn T, Trzaskoma B (2013). Concomitant asthma medica- tions in moderate-to-severe allergic asthma treated with omalizumab. Respir Med.

[89] Tzortzaki EG, Georgiou A, Kampas D, Lemessios M, Markatos M, Adamidi T (2012). Long-term omalizumab treatment in severe allergic asthma: the south- eastern Mediterranean “real-life” experience. Pulm Pharmacol Ther.

[90] Vennera Mdel C, Perez De Llano L, Bardagí S, Ausin P, Sanjuas C, Gonzalez H (2012). Omalizumab therapy in severe asthma: experience from the Spanish registryesome new approaches. J Asthma.

[91] Abonia JP, Putnam PE (2011). Mepolizumab in eosinophilic disorders. Expert Rev Clin Immunol.

[92] Chupp GL, Bradford ES, Albers FC, Bratton DJ, Wang-Jairaj J, Nelsen LM, Trevor JL, Magnan A, Ten Brinke A (2017). Efficacy of mepolizumab add-on therapy on health-related quality of life and markers of asthma control in severe eosinophilic asthma (MUSCA): a randomised, double-blind, placebo-controlled, parallel-group, multicentre, phase 3b trial. Lancet Respir Med.

[93] Powell C, Milan SJ, Dwan K, Bax L, Walters N. Mepolizumab versus placebo for asthma. Cochrane Database Syst Rev. 2015;27(7):CD010834. 10.1002/14651858.CD010834.pub2 26214266

[94] Haldar P, Brightling CE, Hargadon B, Gupta S, Monteiro W, Sousa A, Marshall RP, Bradding P, Green RH, Wardlaw AJ, Pavord ID (2009). Mepolizumab and exacerbations of refractory eosinophilic asthma. N Engl J Med.

[95] Nair P, Pizzichini MM, Kjarsgaard M, Inman MD, Efthimiadis A, Pizzichini E, Hargreave FE, O’Byrne PM (2009). Mepolizumab for prednisone-dependent asthma with sputum eosinophilia. N Engl J Med.

[96] Pavord ID, Korn S, Howarth P, Bleecker ER, Buhl R, Keene ON, Ortega H, Chanez P (2012). Mepolizumab for severe eosinophilic asthma (DREAM): a multicentre, double-blind, placebo-controlled trial. Lancet.

[97] Bel EH, Wenzel SE, Thompson PJ, Prazma CM, Keene ON, Yancey SW, Ortega HG, Pavord ID, SIRIUS Investigators (2014). Oral glucocorticoid-sparing effect of mepolizumab in eosinophilic asthma. N Engl J Med.

[98] Ortega HG, Liu MC, Pavord ID, Brusselle GG, FitzGerald JM, Chetta A, Humbert M, Katz LE, Keene ON, Yancey SW, Chanez P, Investigators MENSA (2014). Mepolizumab treatment in patients with severe eosinophilic asthma. N Engl J Med.

[99] Hom S, Pisano M (2017). Reslizumab (Cinqair): an Interleukin-5 antagonist for severe asthma of the eosinophilic phenotype. P T.

[100] Castro M, Mathur S, Hargreave F, Boulet LP, Xie F, Young J, Wilkins HJ, Henkel T, Nair P (2011). Res-5-0010 study group. Reslizumab for poorly controlled, eosinophilic asthma: a randomized, placebo-controlled study. Am J Respir Crit Care Med.

[101] Castro M, Zangrilli J, Wechsler ME, Bateman ED, Brusselle GG, Bardin P, Murphy K, Maspero JF, O’Brien C, Korn S (2015). Reslizumab for inadequately controlled asthma with elevated blood eosinophil counts: results from two multicentre, parallel, double-blind, randomised, placebo-controlled, phase 3 trials. Lancet Respir Med.

[102] Corren J, Weinstein S, Janka L, Zangrilli J, Garin M (2016). Phase 3 study of Reslizumab in patients with poorly controlled asthma: effects across a broad range of eosinophil counts. Chest.

[103] Laviolette M, Gossage DL, Gauvreau G, Leigh R, Olivenstein R, Katial R, Busse WW, Wenzel S, Wu Y, Datta V, Kolbeck R, Molfino NA (2013). Effects of benralizumab on airway eosinophils in asthmatic patients with sputum eosinophilia. J Allergy Clin Immunol.

[104] Hilvering B, Xue L, Pavord ID (2015). Evidence for the efficacy and safety of anti-interleukin-5 treatment in the management of refractory eosinophilic asthma. Ther Adv Respir Dis.

[105] Kolbeck R, Kozhich A, Koike M, Peng L, Andersson CK, Damschroder MM, Reed JL, Woods R, Dall’acqua WW, Stephens GL, Erjefalt JS, Bjermer L, Humbles AA, Gossage D, Wu H, Kiener PA, Spitalny GL, Mackay CR, Molfino NA, Coyle AJ (2010). MEDI-563, a humanized anti-IL-5 receptor alpha mAb with enhanced antibody-dependent cell-mediated cytotoxicity function. J Allergy Clin Immunol.

[106] Nowak RM, Parker JM, Silverman RA, Rowe BH, Smithline H, Khan F, Fiening JP, Kim K, Molfino NA (2015). A randomized trial of benralizumab, an antiinterleukin 5 receptor α monoclonal antibody, after acute asthma. Am J Emerg Med.

[107] Castro M, Wenzel SE, Bleecker ER, Pizzichini E, Kuna P, Busse WW, Gossage DL, Ward CK, Wu Y, Wang B, Khatry DB, van der Merwe R, Kolbeck R, Molfino NA, Raible DG (2014). Benralizumab, an anti-interleukin 5 receptor α monoclonal antibody, versus placebo for uncontrolled eosinophilic asthma: a phase 2b randomised dose-ranging study. Lancet Respir Med.

[108] FitzGerald JM, Bleecker ER, Nair P, Korn S, Ohta K, Lommatzsch M, Ferguson GT, Busse WW, Barker P, Sproule S, Gilmartin G, Werkström V, Aurivillius M, Goldman M, study investigators CALIMA (2016). Benralizumab, an anti-interleukin-5 receptor α monoclonal antibody, as add-on treatment for patients with severe, uncontrolled, eosinophilic asthma (CALIMA): a randomised, double-blind, placebo-controlled phase 3 trial. Lancet.

[109] Bleecker ER, FitzGerald JM, Chanez P, Papi A, Weinstein SF, Barker P, Sproule S, Gilmartin G, Aurivillius M, Werkström V, Goldman M, SIROCCO study investigators (2016). Efficacy and safety of benralizumab for patients with severe asthma uncontrolled with high-dosage inhaled corticosteroids and long-acting β<sub>2</sub>−agonists (SIROCCO): a randomised, multicentre, placebo-controlled phase 3 trial. Lancet.

[110] Hart TK, Blackburn MN, Brigham-Burke M, Dede K, Al-Mahdi N, Zia-Amirhosseini P, Cook RM (2002). Preclinical efficacy and safety of pascolizumab (SB 240683): a humanized anti-interleukin-4 antibody with therapeutic potential in asthma. Clin Exp Immunol.

[111] Slager RE, Otulana BA, Hawkins GA, Yen YP, Peters SP, Wenzel SE, Meyers DA, Bleecker ER (2012). IL-4 receptor polymorphisms predict reduction in asthma exacerbations during response to an anti-IL-4 receptor α antagonist. J Allergy Clin Immunol.

[112] Wenzel S, Ford L, Pearlman D, Spector S, Sher L, Skobieranda F, Wang L, Kirkesseli S, Rocklin R, Bock B, Hamilton J, Ming JE, Radin A, Stahl N, Yancopoulos GD, Graham N, Pirozzi G (2013). Dupilumab in persistent asthma with elevated eosinophil levels. N Engl J Med.

[113] Wenzel S, Castro M, Corren J, Maspero J, Wang L, Zhang B, Pirozzi G, Sutherland ER, Evans RR, Joish VN, Eckert L, Graham NM, Stahl N, Yancopoulos GD, Louis-Tisserand M, Teper A (2016). Dupilumab efficacy and safety in adults with uncontrolled persistent asthma despite use of medium-to-high-dose inhaled corticosteroids plus a long-acting β2 agonist: a randomised double-blind placebo-controlled pivotal phase 2b dose-ranging trial. Lancet.

[114] Hua F, Ribbing J, Reinisch W, Cataldi F, Martin S (2015). A pharmacokinetic comparison of anrukinzumab, an anti- IL-13 monoclonal antibody, among healthy volunteers, asthma and ulcerative colitis patients. Br J Clin Pharmacol.

[115] Hanania NA, Noonan M, Corren J, Korenblat P, Zheng Y, Fischer SK, Cheu M, Putnam WS, Murray E, Scheerens H, Holweg CT, Maciuca R, Gray S, Doyle R, McClintock D, Olsson J, Matthews JG, Yen K (2015). Lebrikizumab in moderate-to-severe asthma: pooled data from two randomised placebo-controlled studies. Thorax.

[116] Corren J, Lemanske RF, Hanania NA, Korenblat PE, Parsey MV, Arron JR, Harris JM, Scheerens H, Wu LC, Su Z, Mosesova S, Eisner MD, Bohen SP, Matthews JG (2011). Lebrikizumab treatment in adults with asthma. N Engl J Med.

[117] Scheerens H, Arron JR, Zheng Y, Putnam WS, Erickson RW, Choy DF, Harris JM, Lee J, Jarjour NN, Matthews JG (2014). The effects of lebrikizumab in patients with mild asthma following whole lung allergen challenge. Clin Exp Allergy.

[118] Noonan M, Korenblat P, Mosesova S, Scheerens H, Arron JR, Zheng Y, Putnam WS, Parsey MV, Bohen SP, Matthews JG (2013). Dose-ranging study of lebrikizumab in asthmatic patients not receiving inhaled steroids. J Allergy Clin Immunol.

[119] Brightling CE, Chanez P, Leigh R, O’Byrne PM, Korn S, She D, May RD, Streicher K, Ranade K, Piper E (2015). Efficacy and safety of tralokinumab in patients with severe uncontrolled asthma: a randomised, double-blind, placebo-controlled, phase 2b trial. Lancet Respir Med.

[120] Piper E, Brightling C, Niven R, Oh C, Faggioni R, Poon K, She D, Kell C, May RD, Geba GP, Molfino NA (2013). A phase II placebo-controlled study of tralokinumab in moderate-to-severe asthma. Eur Respir J.

[121] Parker JM, Glaspole IN, Lancaster LH, Haddad TJ, She D, Roseti SL, Fiening JP, Grant EP, Kell CM, Flaherty KR. A Phase 2 randomized controlled study of Tralokinumab in subjects with idiopathic pulmonary fibrosis. Am J Respir Crit Care Med. 2018;197:94-103. 10.1164/rccm.201704-0784OC 28787186

[122] Corren J, Parnes JR, Wang L, Mo M, Roseti SL, Griffiths JM, van der Merwe R (2017). Tezepelumab in adults with uncontrolled asthma. N Engl J Med.

